# Identification of uterine leiomyoma-specific marker genes based on DNA methylation and their clinical application

**DOI:** 10.1038/srep30652

**Published:** 2016-08-08

**Authors:** Shun Sato, Ryo Maekawa, Yoshiaki Yamagata, Isao Tamura, Lifa Lee, Maki Okada, Kosuke Jozaki, Hiromi Asada, Hiroshi Tamura, Norihiro Sugino

**Affiliations:** 1 Department of Obstetrics and Gynecology, Yamaguchi University Graduate School of Medicine, Minamikogushi 1-1-1, Ube, 755-8505 Japan

## Abstract

Differential diagnosis of uterine leiomyomas and leiomyosarcomas is needed to determine whether the uterus can be retained. Therefore, biomarkers for uterine leiomyomas, and reliable and objective diagnostic methods have been desired besides the pathological diagnosis. In the present study, we identified 12 genes specific to uterine leiomyomas based on DNA methylation. Using these marker genes specific to uterine leiomyomas, we established a hierarchical clustering system based on the DNA methylation level of the marker genes, which could completely differentiate between uterine leiomyomas and normal myometrium. Furthermore, our hierarchical clustering system completely discriminated uterine cancers and differentiated between uterine leiomyosarcomas and leiomyomas with more than 70% accuracy. In conclusion, this study identified DNA methylation-based marker genes specific to uterine leiomyomas, and our hierarchical clustering system using these marker genes was useful for differential diagnosis of uterine leiomyomas and leiomyosarcomas.

Uterine leiomyomas are tumors that are derived from uterine smooth muscle cells and are most common in gynecologic neoplasms^1^. More than 30% of reproductive-age women suffer from uterine leiomyomas^1^. Although uterine leiomyomas are benign, they cause severe pelvic pain, menorrhagia, dysmenorrhea, anemia, infertility and miscarriage^1^,^2^. Therefore, the quality of life of women with uterine leiomyomas is significantly impaired. Surgery has long been the main mode of therapy for uterine leiomyomas. For many women who have completed childbearing, hysterectomy is an attractive option to eliminate such problems. However, in recent years, the average age of marriage is increasing due to changes in women’s lifestyle. Women with uterine leiomyomas who wish to retain the uterus for future pregnancies are also increasing in number. For such women, myomectomy is the recommended surgery.

Uterine smooth muscle neoplasms have malignant uterine leiomyosarcomas in addition to benign uterine leiomyomas. Uterine leiomyosarcomas are high-grade tumors whose 5-year survival rate is less than 50%, and metastasize to the lungs or liver in the early stages^3^,^4^. The risk factors of uterine leiomyosarcomas are not clear. Conventional chemotherapy and radiotherapy have little effect at prolonging survival, leaving hysterectomy as the only option^3^,^5^,^6^,^7^. Because uterine leiomyomas and leiomyosarcomas occur in similar location and have similar shaped tumors, differentiating them can be difficult. Uterine leiomyosarcomas are diagnosed based on three pathological findings using light microscopy; increased activity of mitosis, cytologic atypia and the presence of coagulative necrosis^8^,^9^. Diagnosis becomes difficult when the diagnostic features are inconsistent. It has been reported that the uterine smooth muscle neoplasms diagnosed as benign leiomyomas metastasize in rare cases^10^,^11^. Such neoplasms can be atypical leiomyomas, cellular leiomyomas or leiomyosarcomas^9^,^12^. Thus, there is a need for differential diagnosis of uterine leiomyomas and leiomyosarcomas in addition to the pathological diagnosis, especially for women who wish to retain the uterus for future pregnancies.

To elucidate the molecular pathogenesis of uterine leiomyomas, researchers have searched for leiomyoma-specific biomarkers. Somatic mutations of *Mediator complex subunit 12* (*MED12*) have been recently reported as reliable biomarkers for uterine leiomyomas^13^. *MED12* mutations were detected in approximately 70% of uterine leiomyoma samples. On the other hand, uterine leiomyosarcomas have been immunohistochemically identified with several biomarkers including PDGFRA, WT1, GNRHR, P53, BCL2, ESR, and PGR^14^,^15^,^16^. They have also been identified by mutations of tumorigenesis-related genes such as *PTEN* and *P53*^12^ and by attenuation of protein expression of LMP2^17^,^18^. LMP2 immunostaining has also been used to distinguish uterine leiomyomas and leiomyosarcomas^17^,^18^. However, those biomarkers are not still used practically for differential diagnosis of uterine leiomyomas and leiomyosarcomas.

Irreversible changes to cell phenotypes may arise from epigenetic mutations (epimutations) as well as mutations. Uterine leiomyomas are thought to be an epimutation-related disease because acquired factors such as obesity, meat consumption, hypertension, a history of pelvic inflammatory diseases place women at greater risk for uterine leiomyomas^19^,^20^,^21^. In fact, genome-wide DNA methylation analyses (DNA methylome) by Navarro *et al*.^22^ and ourselves^23^,^24^,^25^ have demonstrated that uterine leiomyomas have an aberrant DNA methylation profile, suggesting that DNA methylation plays a key role in the pathogenesis of uterine leiomyomas^26^. DNA methylation is a major type of epigenetic mark. DNA methylation profiles define and distinguish between each type of normal cell^27^,^28^, and thus have been used to characterize abnormal cells^29^,^30^. Interestingly, by using a principal component analysis, we previously found clear differences in DNA methylation between uterine leiomyomas and normal myometrium, but did not find clear differences in mRNA expression^25^. Thus, profiling by DNA methylation is better at defining cell identity than profiling by mRNA expression. These results prompted us to search for marker genes specific to uterine leiomyomas based on DNA methylation.

We previously reported the 120 aberrantly methylated genes specific to uterine leiomyomas using DNA methylome and transcriptome data^25^. In the present study, we first identified 12 of these120 genes as maker genes by comparing DNA methylation levels in multiple paired samples of the leiomyoma and adjacent normal myometrium. Then, we attempted to construct a hierarchical clustering system based on DNA methylation levels of the marker genes for differentiating uterine leiomyomas and normal myometrium. We further examined whether this hierarchical clustering system based on DNA methylation levels can be used to discriminate uterine cancers or uterine leiomyosarcomas from uterine leiomyomas.

## Results

### Identification of leiomyoma-specific marker genes

**Figure 1:**
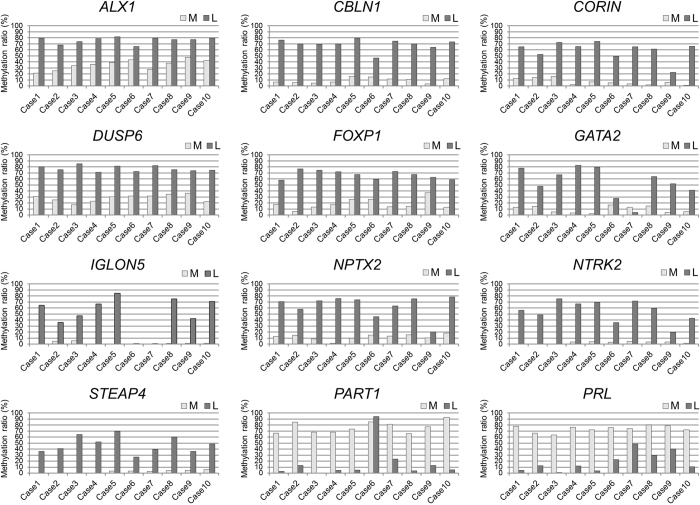
Methylation levels of 12 leiomyoma-specific marker genes in 10 paired samples of uterine leiomyomas and adjacent normal myometrium. The gray and black bars indicate the percentage of DNA methylation in the myometrium (M) and uterine leiomyoma (L) specimens, respectively. DNA methylation was evaluated by COBRA.

Of the 120 aberrantly methylated genes specific to uterine leiomyomas^25^, we first selected 33 genes that have the necessary restriction enzyme sites for the combined bisulfite restriction analysis (COBRA) (Table 1) and confirmed their DNA methylation levels with 10 paired samples of the leiomyoma and adjacent normal myometrium. Genes that showed hypermethylation or hypomethylation in at least 70% of the leiomyoma specimens were defined as leiomyoma-specific marker genes. Twelve of the 33 genes satisfied this criterion. They included 10 hypermethylated genes (*ALX1, CBLN1, CORIN, DUSP6, FOXP1, GATA2, IGLON5, NPTX2, NTRK2* and *STEAP4*) and 2 hypomethylated genes (*PART1* and *PRL*) (Fig. 1).

### DNA methylation-based hierarchical clustering with leiomyoma-specific marker genes

**Figure 2:**
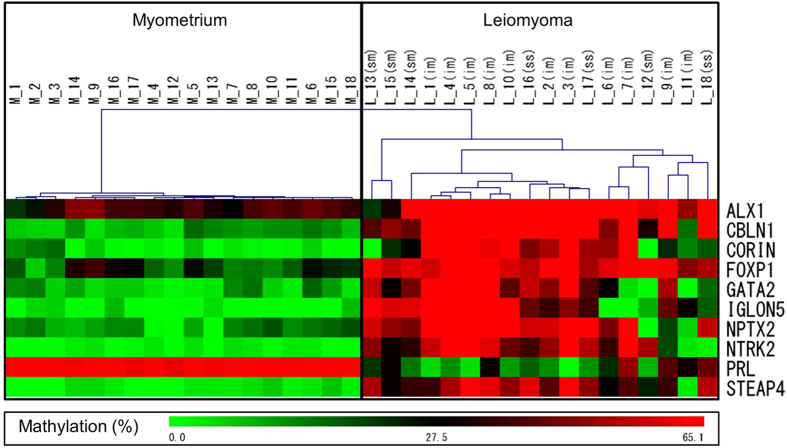
Hierarchical clustering of 18 paired samples of leiomyoma and normal myometrium based on DNA methylation level of leiomyoma-specific marker genes. DNA methylation levels of the 10 leiomyoma-specific marker genes (*ALX1, CBLN1, CORIN, FOXP1, GATA2, IGLON5, NPTX2, NTRK2, PRL* and *STEAP4*) were measured by COBRA method in 18 paired specimens of uterine leiomyomas (L-1 to -18) and adjacent normal myometrium (M-1 to -18). Samples were clustered according to the similarity of their gene methylation profiles (hierarchical clustering). Uterine leiomyomas were classified into three types; intramural (im), submucosal (sm) and subserosal (ss) leiomyoma. Each type of uterine leiomyomas is indicated as im, sm, and ss.

DNA methylation levels of the 12 leiomyoma-specific marker genes were measured in the 18 paired samples of the leiomyoma (L-1 to -18) and adjacent normal myometrium (M-1 to -18) by COBRA method. Among the 12 leiomyoma-specific marker genes, we attempted to select the most appropriate combination of the marker genes that best differentiate between leiomyomas and normal myometrium based on DNA methylation level of the marker gene. For this purpose, we tested several combinations of the marker genes using the hierarchical clustering based on the gene methylation profile of the 18 paired samples. Samples were clustered according to the similarity of their gene methylation profiles. Among the 12 leiomyoma-specific marker genes, the combination of the 10 marker genes (*ALX1, CBLN1, CORIN, FOXP1, GATA2, IGLON5, NPTX2, NTRK2, PRL* and *STEAP4*) completely differentiated between the leiomyoma and normal myometrium in the hierarchical clustering based on the DNA methylation profiles (Fig. 2).

Uterine leiomyomas are classified as intramural (im), submucosal (sm) and subserosal (ss) based on their location^1^,^2^. The DNA methylation profiles of the marker genes varied among the three types of the leiomyoma, and thus were not specific to any of the types of uterine leiomyomas (Fig. 2). This hierarchical clustering system with the 10 marker genes was utilized in the following investigations.

### Hierarchical clustering of solitary and multifocal leiomyomas

**Figure 3:**
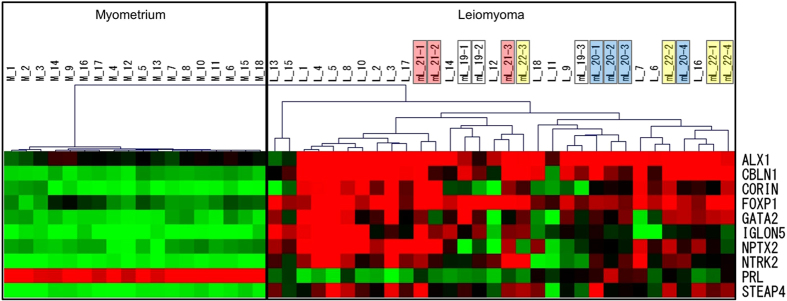
Hierarchical clustering of solitary and multifocal leiomyomas. In four additional patients with multifocal leiomyomas, three or four leiomyoma nodules were obtained from an individual. DNA methylation levels of the 10 leiomyoma-specific marker genes were measured in each of three or four leiomyoma nodules (a total of 14 nodules). Then, the DNA methylation profile of each sample (mL-19-1 to -3, mL-20-1 to -4, mL-21-1 to -3, mL-22-1 to -4) above were subjected to the hierarchical clustering with the 18 paired samples of the leiomyoma (L-1 to -18) and normal myometrium (M-1 to -18) in Fig. 2.

More than half of all cases of uterine leiomyomas are multifocal leiomyomas. The 18 cases examined in this study (Fig. 2) had solitary leiomyomas. Therefore, we investigated whether the DNA methylation profiles of the marker genes are different in solitary and multifocal leiomyomas. In four additional patients with multifocal leiomyomas, DNA methylation levels of the 10 leiomyoma-specific marker genes were measured in each of three or four leiomyoma nodules (a total of 14 nodules). Then, the DNA methylation profile of each sample was subjected to the hierarchical clustering with the 18 paired samples of the leiomyoma and normal myometrium in Fig. 2 (Fig. 3). The multifocal leiomyomas (new 14 specimens) did not make a separate cluster from the solitary leiomyomas, suggesting that there was no specificity in DNA methylation profiles of the marker genes between the solitary and multifocal leiomyomas (Fig. 3). In addition, DNA methylation profiles differed among the leiomyoma nodules in a patient with multifocal leiomyomas (Fig. 3).

### *MED12* somatic mutations analysis and hierarchical clustering in the leiomyoma specimens

**Figure 4:**
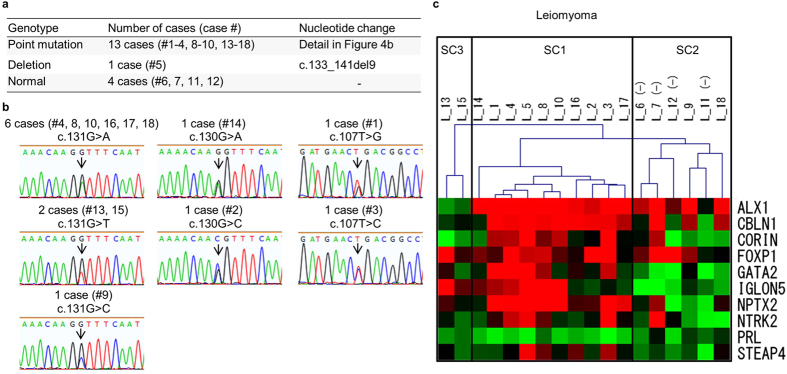
Analysis of somatic mutations of *MED12* gene and hierarchical clustering in the leiomyoma specimens. (**a**) Summary of the *MED12* mutation analysis in 18 leiomyoma specimens used in the study. (**b**) Sequencing chromatogram showing the point mutation in *MED12* in the leiomyoma specimens. Each point mutation is shown on the chromatogram. Mutated bases are indicated by arrows. (**c**) The hierarchical clustering of 18 uterine leiomyoma samples (L-1 to -18). DNA methylation profiles of the leiomyoma samples were classified into the three sub-clusters (SC1, SC2 and SC3). The leiomyoma specimens without *MED12* mutations are shown as (-).

Fourteen (78%) of the 18 leiomyoma specimens had somatic mutations (a single nucleotide mutation in 13 specimens and a deletion mutation in 1 specimen) in *MED12* (Fig. 4a,b). All of the single nucleotide mutations were at hot spots of uterine leiomyomas (positions 107, 130 and 131) of the coding region (Fig. 4b)^13^.

As shown in Fig. 2, DNA methylation profiles of the marker genes were highly homogeneous among the 18 normal myometrium samples in the hierarchical clustering system. On the other hand, DNA methylation profiles of the leiomyoma samples were heterogeneous, but it was possible that they are classified into the three sub-clusters (SC1, SC2 and SC3 in Fig. 4c). Interestingly, the 4 leiomyoma specimens without *MED12* mutations were included in the same sub-cluster, SC2 (Fig. 4c).

### Hierarchical clustering of uterine cancers

**Figure 5:**
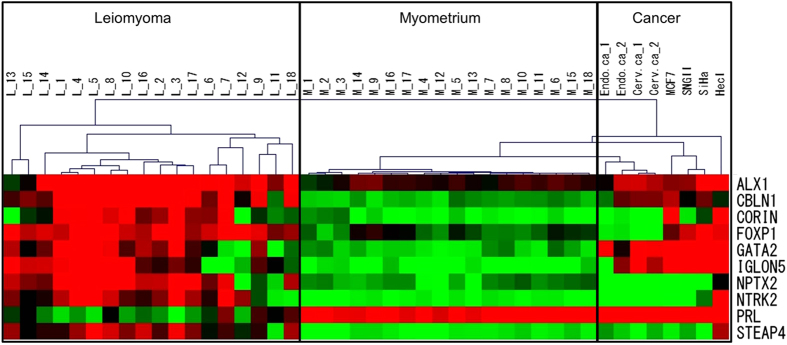
Hierarchical clustering of uterine leiomyomas and uterine cancers. DNA methylation levels of the 10 leiomyoma-specific marker genes were measured in 2 cases of endometrial cancer (Endo.ca-1 and -2), 2 cases of cervical cancer (Cerv.ca-1 and -2), 2 cell lines of endometrial cancer (HecI and SNGII), a cervical cancer cell line (SiHa), and a breast cancer cell line (MCF7). Then, the DNA methylation profile of each sample above were subjected to the hierarchical clustering with the 18 paired samples of the leiomyoma (L-1 to -18) and normal myometrium (M-1 to -18) in Fig. 2.

Next, we examined whether our hierarchical clustering system can discriminate uterine cancers from uterine leiomyomas. DNA methylation levels of the10 marker genes were measured in endometrial cancers (Endo. ca-1 and -2), cervical cancers (Cerv. ca-1 and -2), two endometrial cancer cell lines (HecI and SNGII), a cervical cancer cell line (SiHa), and a breast cancer cell line (MCF7). Then, the DNA methylation profile of each sample was subjected to the hierarchical clustering with the 18 paired samples of the leiomyoma and normal myometrium in Fig. 2. All of the uterine cancer samples and cell lines were classified into a separate cluster (Fig. 5).

### Hierarchical clustering of leiomyomas and leiomyosarcomas

**Figure 6:**
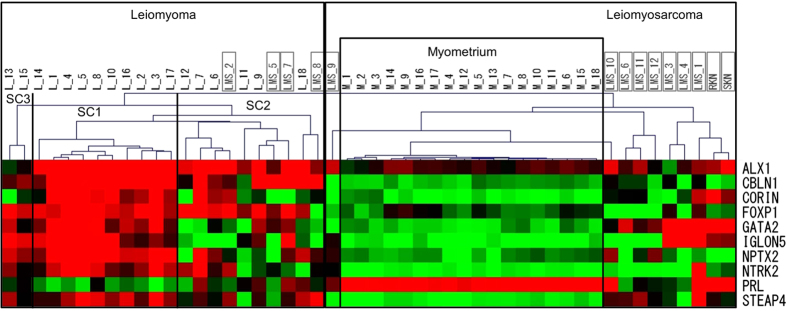
Hierarchical clustering of uterine leiomyomas and leiomyosarcomas. DNA methylation levels of the 10 leiomyoma-specific marker genes were measured in 12 uterine leiomyosarcoma specimens (LMS-1 to -12), and a uterine sarcoma cell line (SKN), and an ovarian sarcoma cell line (RKN). Then, the DNA methylation profile of each sample of leiomyosarcomas above (14 samples; gray boxes) were subjected to the hierarchical clustering with the 18 paired samples of the leiomyoma (L-1 to -18) and normal myometrium (M-1 to -18) in Fig. 2. Cases #LMS-1, -3, -4, -6, -9, -10, -11, -12, SKN, and RKN were classified into a separate cluster (Leiomyosarcoma). Cases #LMS-2, -5, -7, and -8 were classified into the leiomyoma group.

We further investigated whether our hierarchical clustering system can differentiate between uterine leiomyomas and uterine leiomyosarcomas. DNA methylation levels of the 10 marker genes were measured in 12 cases of the uterine leiomyosarcoma specimens (LMS-1 to -12, grey boxes in Fig. 6), a uterine leiomyosarcoma cell line (SKN, grey box) and an ovarian leiomyosarcoma cell line (RKN, grey box). Then, the DNA methylation profile of each sample was subjected to the hierarchical clustering with the 18 paired samples of the leiomyoma and normal myometrium in Fig. 2. Eight out of 12 uterine leiomyosarcoma specimens (LMS-1, -3, -4, -6, -9, -10 -11, and -12) and the two leiomyosarcoma cell lines (RKN and SKN) formed a cluster (Fig. 6). The other four uterine leiomyosarcoma specimens (LMS-2, -5, -7 and -8) clustered in the SC2 sub-cluster of the leiomyoma group (Fig. 6). Thus, 10 (71%) out of the 14 leiomyosarcomas clustered together, indicating that this hierarchical clustering system can differentiate between uterine leiomyosarcomas and uterine leiomyomas with approximately 70% accuracy.

*MED12* mutations were analyzed in the 12 uterine leiomyosarcoma samples. Only one sample (LMS-4) had a point mutation (Supplementary Figure S1).

## Discussion

Uterine leiomyoma is a monoclonal tumor derived from a single cell^31^,^32^. Therefore, cytogenetic abnormalities and somatic mutations are likely to become useful biomarkers. Cytogenetic abnormalities in uterine leiomyomas include trisomy of chromosome 12, translocation between chromosomes 12 and 14, inversion or translocation between chromosomes 6 and 10, and deletion of chromosomes 3 and 7^33^,^34^. Because of their low frequency, these abnormalities are not useful biomarkers. On the other hand, somatic mutations in the vicinity of exon 2 of the *MED12* gene were detected in about 70% of uterine leiomyomas^13^. However, *MED12* mutations cannot completely differentiate between uterine leiomyomas and normal myometrium. It is interesting to note that irreversible changes to cell phenotypes arise from epimutations as well as mutations. A number of genomic loci in uterine leiomyomas have epimutations such as changes in DNA methylation, suggesting that these mutations are involved in the development of uterine leiomyomas^22^,^23^,^24^,^25^,^26^. Since the DNA methylation profile is specific for each cell type, the profiles can distinguish between normal and abnormal cells^27^,^28^,^29^,^30^. Our previous principal component analysis showed that profiling by DNA methylation is better at defining cell identity than profiling by mRNA expression^25^. In this study, we identified the marker genes specific to uterine leiomyomas based on DNA methylation. The present study is the first report to show that the hierarchical clustering system with leiomyoma-specific marker genes can differentiate between uterine leiomyomas and normal myometrium.

For the clinical application of leiomyoma-specific marker genes, we utilized the hierarchical clustering system for differential diagnosis between uterine leiomyomas and uterine leiomyosarcomas. Interestingly, our hierarchical clustering system discriminated leiomyosarcomas from leiomyomas with more than 70% accuracy. We expect our hierarchical clustering system to become a reliable and objective diagnostic method. The expression of LMP2 protein was found to be attenuated in 85% of specimens of uterine leiomyosarcomas, and LMP2 immunostaining was shown to be useful for differential diagnosis of uterine leiomyomas and leiomyosarcomas^17^,^18^. A combination of LMP2 immunostaining and our hierarchical clustering system should provide more reliable criteria for differential diagnosis of uterine leiomyomas and leiomyosarcomas.

Uterine leiomyomas are classified into three types (intramural, submucosal and subserosal) based on their location^1^,^2^. They can also be classified as solitary and multifocal leiomyomas. It is unclear whether the different types of uterine leiomyomas differ at the molecular level. Our hierarchical clustering system showed that the DNA methylation profiles were not specific to any of the types of uterine leiomyomas. It is interesting to note that DNA methylation profiles differed among the leiomyoma nodules in a patient with multifocal leiomyomas, supporting the consensus that uterine leiomyomas are monoclonal tumors derived from a single cell^31^,^32^.

DNA methylation profiles of the marker genes were highly homogeneous in normal myometrium by our hierarchical clustering system while they were heterogeneous in uterine leiomyomas. This suggests that uterine leiomyomas do not always occur and develop in the same way or by the same mechanism, indicating that complex networks by genetic and epigenetic factors are involved in the pathogenesis of uterine leiomyomas^26^,^35^,^36^,^37^,^38^. In this study, DNA methylation profiles of the leiomyoma were classified into the three sub-clusters (SC1, SC2 and SC3 in Fig. 4c). All of the 4 leiomyoma specimens without *MED12* mutations were included in the same cluster, SC2. *MED12* mutations have been reported to be involved in the development of uterine leiomyomas^39^. The molecular pathogenesis of uterine leiomyomas in SC2 may be different from that of leiomyoma with *MED12* mutations. Thus, our hierarchical clustering system may provide an unprecedented opportunity to reveal subtypes of uterine leiomyomas. All of the uterine leiomyosarcoma specimens that were clustered into the leiomyoma group were included in SC2, indicating that the DNA methylation profiles of the marker genes are similar to the marker gene profiles of uterine leiomyomas without *MED12* mutations. Furthermore, all of the leiomyosarcoma samples (LMS-2, -5, -7 and -8) clustered in the SC2 sub-cluster of the leiomyoma group had no *MED12* mutations (Supplementary Figure S1). Since DNA methylation profiles are specific to cell types^27^,^28^, some cases of uterine leiomyosarcomas may be derived from uterine leiomyoma cells without *MED12* mutations. This supports the previous reports that uterine leiomyosarcomas may arise from preexisting leiomyomas^40^,^41^,^42^. Thus, our results might shed light on the differences in the pathogenesis of the subtype of leiomyomas and provide the clue of the origin of the leiomyosarcomas clustered into the leiomyoma group.

Our hierarchical clustering system based on the DNA methylation level of the marker genes may be useful for differential diagnosis between uterine leiomyomas and leiomyosarcomas. It is beneficial especially for women who wish to retain the uterus for future pregnancies. Furthermore, it is difficult at present to evaluate whether smooth muscle cells were transformed to leiomyoma cells in *in vitro* experiments. Our hierarchical clustering system may help to evaluate the cell identity, and also contributes to elucidating the molecular pathogenesis of uterine leiomyomas.

## Methods

### Ethical statement

This study was approved by the Institutional Review Board of Yamaguchi University Graduate School of Medicine. Informed consent was obtained from the patients before the collection of any samples. All experiments handling human tissues were performed in accordance with Tenets of the Declaration of Helsinki.

### Tissue samples and cell culture

Paired specimens of uterine leiomyoma and adjacent normal myometrium were obtained from 22 Japanese women who underwent hysterectomy at the Department of Obstetrics and Gynecology at Yamaguchi University Hospital. Among the 22 women, eighteen women had a single leiomyoma nodule and four women had multiple nodules. None of the women had received previous treatment with sex steroid hormones or gonadotropin releasing hormone analogs. Tissue samples of uterine leiomyosarcoma, endometrial cancer, and cervical cancer were also obtained from five Japanese women at the time of surgery at the Department of Obstetrics and Gynecology at Yamaguchi University Hospital. None of the women had received anticancer chemotherapy or irradiation therapy before the operation. Dissected specimens were immediately immersed in liquid nitrogen and stored at −80 °C until the DNA isolation. Two frozen tissue samples of uterine leiomyosarcomas were provided from Shinshu University. Three DNA samples and six formalin fixed paraffin embedded (FFPE) tissues of uterine leiomyosarcomas were provided from Tohoku University.

Human cervical epidermoid carcinoma cell line (SKGII), human tumor cell line from uterine Leiomyosarcoma (SKN) and human tumor cell line from an ovarian myosarcoma (RKN) were cultured in Ham’s F12 medium (Wako, Osaka, Japan) supplemented with 10% fetal bovine serum (FBS). Human endometrial adenocarcimona cell line (HecI) and human uterine cervical SCC cell line (SiHa) were cultured in Eagle’s minimum essential medium (Sigma-Aldrich, Tokyo, Japan) supplemented with 10% FBS. Human metastatic mammary carcinoma cell line (MCF7) was cultured in Eagle’s minimum essential medium supplemented with non-essential amino acids (Gibco BRL, Rockville, MD, USA), 1 mM sodium pyruvate (Gibco BRL) and 10% FBS.

### Genomic DNA isolation

DNA from the tissue specimens was isolated as reported previously^43^. In brief, the genomic DNA was isolated by treatment with proteinase K (Qiagen, Hilden, Germany), followed by phenol/chloroform extraction and ethanol precipitation. DNA from the FFPE specimens and cell lines were isolated using QIAamp DNA FFPE tissue kit (Qiagen) and Allprep DNA/RNA mini kit (Qiagen), respectively, according to the manufacturer’s instructions.

### Combined Bisulfite Restriction Analysis (COBRA)

DNA methylation levels were evaluated by COBRA (Supplemental Figure S2) as we previously reported^25^,^44^. In brief, sodium bisulfite treatment was performed using EpiTect Bisulfite kit (Qiagen) according to the conditions as follows: 95 °C for 5 min, 65 °C for 85 min, 95 °C for 5 min and 65 °C for 175 min. After sodium bisulfite treatment, PCR was performed using one unit of Biotaq HS DNA polymerase (Bioline, London, UK) and the primer sets shown in Table 2 under the thermocycling conditions (35 to 38 cycles of 95 °C for 30 sec, 60 °C for 30 sec, and 72 °C for 30 sec, with an initial step of 95 °C for 10 min and a final step of 72 °C for 7 min). A part of the PCR product was digested with the restriction enzyme *Taq*I (Takara, Tokyo, Japan) or *Hpy*CH4IV (New England Biolabs, Ipswich, MA). The treated PCR product was electrophoresed by 3% agarose gel. PCR products from methylated DNA and unmethylated DNA are digested and undigested by the treatment with the restriction enzyme. The intensity of the signals of the digested and undigested PCR products was measured by densitometry. Methylation levels (%) were calculated as the ratio of the digested PCR product in the total PCR product (digested + undigested products) (Supplementary Figure S2). In the study, the aberrant methylation of the loci in the leiomyomas was defined as more than 30% higher or lower methylation ratio compared to that of the corresponding myometrium. The methylation ratio of the 10 leiomyoma-specific marker genes in all samples used in this study was also shown in Supplementary Table S1.

### Hierarchical clustering analysis

The methylation ratio of the 10 marker genes specific to the uterine leiomyoma in each sample, which was measured by COBRA method, was subjected to the hierarchical clustering analysis. Hierarchical clustering was performed by the open resource software MultiExperiment Viewer (MeV; http://www.tm4.org/mev.html)^45^. In this study, Pearson correlation and Average linkage clustering were used to calculate the matrix distance and to build clusters, respectively.

### *MED12* exon2 genomic sequencing

*MED12* in the leiomyoma and leiomyosarcoma specimens was sequenced as described previously with some modifications^13^,^44^. In brief, genomic PCR was performed using 1.25 units of PrimeSTAR GXL DNA polymerase (Takara) and a primer set as follows: 5′-GCCCTTTCACCTTGTTCCTT-3′ and 5′-TGTCCCTATAAGTCTTCCCAACC-3′ under the thermoscycling conditions (35 cycles of 98 °C for 10 sec, 60 °C for 15 sec, and 68 °C for 45 sec). The amplified PCR products were purified using QIAquick PCR purification kit (Qiagen), and sequenced by 3130xl Genetic Analyzer. The obtained sequence chromatograms were analyzed manually.

## Additional Information

**How to cite this article**: Sato, S. *et al*. Identification of uterine leiomyoma-specific marker genes based on DNA methylation and their clinical application. *Sci. Rep*. **6**, 30652; doi: 10.1038/srep30652 (2016).

## Supplementary Material

Supplementary Information

## Figures and Tables

**Table 1:** Candidates for the leiomyoma-specific marker genes subjected to COBRA.

ID	Gene name	Amplified region (UCSC hg19)	Product size (bp)	No. of CpGs
1	*ALX1*	chr12: 85673057-85673382	326	19
2	*CBLN1*	chr16: 49311435-49311838	404	24
3	*CCDC68*	chr18: 52626491-52626903	413	39
4	*CD74*	chr5: 149792162-149792444	283	11
5	*CORIN*	chr4: 47839848-47840269	422	20
6	*CYP1B1*	chr2: 38300472-38300814	344	11
7	*DAPK1*	chr9: 90113476-90113903	428	33
8	*DUSP6*	chr12: 89744355-89744564	210	6
9	*EFEMP1*	chr2: 56150216-56150531	316	17
10	*EFHD1*	chr2: 233498692-233499129	438	33
11	*ELTD1*	chr1: 79472153-79472521	369	31
12	*FOXP1*	chr3: 71180075-71180444	370	5
13	*GATA2*	chr3: 128204757-128205043	287	12
14	*GRAMD3*	chr5: 125758714-125758973	260	6
15	*GRIN2A*	chr16: 10274346-10274721	376	15
16	*IGLON5*	chr19: 51830135-51830609	475	35
17	*KIF5C*	chr2: 149645634-149646094	461	28
18	*NPTX2*	chr7: 98248945-98249227	283	15
19	*NR5A2*	chr1: 200007489-200007746	258	9
20	*NTRK2*	chr9: 87282683-87282911	229	11
21	*NUAK1*	chr12: 106533813-106534096	283	11
22	*PART1*	chr5: 59783983-59784268	286	10
23	*PLCE1*	chr10: 95848581-95848961	381	6
24	*PRL*	chr6: 22294453-22294794	342	13
25	*SASH1*	chr6: 148869422-148869731	310	12
26	*SPATA18*	chr4: 52942904-52943330	427	26
27	*SPTBN1*	chr2: 54682349-54682676	328	15
28	*STEAP4*	chr7: 87936313-87936586	274	6
29	*TFAP2C*	chr20: 55199951-55200241	291	11
30	*TMEM173*	chr5: 138862276-138862544	269	4
31	*TUFT1*	chr1: 151513348-151513567	220	6
32	*WNT2B*	chr1: 113051737-113052034	298	11
33	*ZFHX4*	chr8: 77593023-77593398	376	24

**Table 2:** PCR primers used for COBRA.

ID	Gene name	Amplified region (UCSC hg19)	Position of analyzed CpG (UCSC hg19)	PCR primers	Product size (bp)	Restriction enzyme Fragment size (bp)
1	*ALX1*	chr12: 85673057-85673382	chr12: 85673201	F: GGAATTAGATGTATTGGTAATTGTTAAT R: AAACCTTTCAAAAACACTAATCCTAAC	326	TaqI 182/144
2	*CBLN1*	chr16: 49311435-49311838	chr16: 49311581	F: TAAGTTTGAAAGGTTGATTTAGTTGTT R: AAATCCCTATCTATCTCTATAAATCCC	404	TaqI 258/146
5	*CORIN*	chr4: 47839848-47840269	chr4: 47840028	F: GGGATTAAGAGGTTATTATATTTGGTTG R: AAAAACTAACTTTCCATATACCCCTAAAAAAT	422	HpyCH4IV 181/241
8	*DUSP6*	chr12:89744355-89744564	chr12: 89744471	F: TTTTTATTTGGGTTGTGTTAAAGATTTT R: TTAATCTCACCTATAAAAAAAATAACCTCAAA	210	TaqI 93/117
12	*FOXP1*	chr3: 71180075-71180444	chr3: 71180142	F: AGATAGGATTGTGATAATTATTTTGGGG R: CTAACTAAACCAAACCCTAACCAAACAC	370	TaqI 303/67
13	*GATA2*	chr3: 128204757-128205043	chr3: 128204927	F: GGGATTGTTATTTTTTATTTTTATGTTTTT R: ACCCTAAAAACCCACTCTCTATATACCC	287	TaqI 171/116
16	*IGLON5*	chr19: 51830135-51830609	chr19: 51830512	F: TGGATAGATTTTATTATTTAGAAGTTGGA R: TACTAATCACAATCAACTATAAACCCCC	475	TaqI 98/377
18	*NPTX2*	chr7: 98248945-98249227	chr7: 98249104	F: TTATAAGTGGTGAAAATTAGGTTATTGTTAGA R: TAACTAAAAACACCAACTCAAAACAAAC	283	TaqI 124/159
20	*NTRK2*	chr9: 87282683-87282911	chr9: 87282747	F: TTTTTTAAGTGTTTTTTAGGGGTTTTTT R: AAACCAACAAATCTATCTCACCACTATC	229	HpyCH4IV 65/164
22	*PART1*	chr5:59783983-59784268	chr5: 59784204	F: GTTTTGGAAAGTTGAAAGGGTTGTATT R: AAAAAAAATAAAAATTACCTCAAAC	286	TaqI 220/65
24	*PRL*	chr6: 22294453-22294794	chr6: 22294711	F: TTTGAGATTTTATGGTATTGTTTTTTTT R: AATATCAAACCTACTCCTATACCAAAAC	342	TaqI 259/83
28	*STEAP4*	chr7: 87936313-87936586	chr7: 87936414	F: AGTTGAGAATTTTAGTAGTTGGAAGGAT R: TACCACCTTTTCTATAAACTAAAACAAAATAA	274	TaqI 102/172
