# Supplementary material for: Identification of uterine leiomyoma-specific marker genes based on DNA methylation and their clinical application
Source: Sci Rep. 2016 Aug 8;6:30652. doi: 10.1038/srep30652 (PMC4976337; doi:10.1038/srep30652)
Supplement: Supplementary Information [file srep30652-s1.pdf]

Supplementary Information

Identification of uterine leiomyoma-specific marker genes based on DNA methylation and their clinical application

Shun Sato, Ryo Maekawa, Yoshiaki Yamagata, Isao Tamura, Lifa Lee, Maki Okada,

Kosuke Jozaki, Hiromi Asada, Hiroshi Tamura and Norihiro Sugino*

Department of Obstetrics and Gynecology, Yamaguchi University Graduate School of Medicine, Minamikogushi 1-1-1, Ube, 755-8505 Japan

Supplementary Table S1. Methylation ratio (%)* of each leiomyoma-specific marker gene in the all samples used in the study

Gene name	M_1	M_2	M_3	M_4	M_5	M_6	M_7	M_8	M_9	M_10	M_11	M_12	M_13	M_14	M_15
ALX1	21.5	25.3	33.7	36	39.4	43.7	27.4	38.2	47.4	42.1	38.6	33.2	32.2	46.9	39.6
CBLN1	6.3	5.5	4.9	6.5	16	14.8	11.7	10.6	3.7	12.1	12.5	3.8	13.2	11.9	12.1
CORIN	12.3	14.3	15.9	2.4	7	5.4	3.4	2	6.1	1.1	1.8	0.7	0.3	0.8	0.5
FOXP1	17.8	5.9	13.4	16.9	26	26.2	13.8	14.5	37.3	12.9	17.4	12.6	20.3	32.6	23.4
GATA2	12.7	14	5.3	3.6	2.4	16.7	12.8	15.2	4.5	5.6	2.8	1.9	3.3	9.8	10.7
IGLON5	0.2	4.7	5.6	0.3	0.7	0.5	0.5	0.8	1.2	1.2	0.5	0.3	4.4	0.8	0.8
NPTX2	12.9	14.6	8.6	1.5	9.8	14.9	13.3	15.3	10.4	18.4	12.6	1.1	0.4	7.6	16.6
NTRK2	0.3	0.4	0.7	3.5	3.7	3.1	4.3	3.3	3.7	1.5	3	0.3	0.5	2.8	0.9
PRL	77.9	66.5	63.6	76.3	72.2	76	74.1	80.3	78.9	72	73.7	60.9	62.9	63.2	78.6
STEAP4	0.4	0.4	0.5	0.4	3.5	3.5	2.9	4.2	5	6.2	2.3	0.4	3.2	4.9	6.9

*Methylation ratio (%) was evaluated by COBRA.

Abbreviations: M; Myometrium, L; Uterine leiomyoma, mL; Multifocal leiomyoma, LMS; Uterine leiomyosarcoma, Endo.ca; Endometrial cancer, Cerv.ca; Cervical cancer.

Gene name	M_16	M_17	M_18	L_1	L_2	L_3	L_4	L_5	L_6	L_7	L_8	L_9	L_10	L_11	L_12
ALX1	37.7	38.4	35.4	79.9	68.1	73.9	79	82	65.8	79.6	77.3	77.2	79.9	49.1	60
CBLN1	7.9	9.1	7.6	76.4	69.9	69.7	70.1	79.7	46.7	74.8	70	64.4	73.5	15.5	32
CORIN	1.3	0.9	1	65.1	52.3	72.7	65.8	74.1	49.7	65.5	61.3	22.9	66	13.4	0.6
FOXP1	30.1	28.3	22.2	57.9	76.7	74.7	72.1	67.2	59.8	72.4	67.5	62.9	58.9	46.9	74.9
GATA2	6.2	6.2	3.6	77.9	47.6	67.1	82.5	79.7	27.6	4.3	64	51.9	41.6	0.9	1
IGLON5	7.8	0.1	7.5	64.7	36.2	47.3	66.9	84.5	0.5	0.8	75.4	42.4	71.1	29.1	8.7
NPTX2	12.9	13	18.5	70.9	58.2	72.3	75.7	73.6	45.6	63.5	75.7	19.9	78.5	5.5	0.2
NTRK2	0.8	2	1.2	56.4	48.7	75.5	66.9	69.7	35.7	71.6	59.8	19.5	43.1	3.2	51.7
PRL	66.2	62.1	71.2	4.7	12.4	0.8	12.1	4.1	22.6	48.6	29.4	40.1	10.7	29.8	8.3
STEAP4	3.1	1	2.5	36.1	41	64.5	52	70	27	39.7	60.3	36.3	48.5	3.1	24

Continued

Gene name	L_13	L_14	L_15	L_16	L_17	L_18	mL_19-1	mL_19-2	mL_19-3	mL_20-1	mL_20-2	mL_20-3	mL_20-4	mL_21-1	mL_21-2
ALX1	21.5	79.5	31.4	69.9	72.1	70.3	59.0	44.9	68.3	82.4	79.3	85.1	86.7	73.5	74.4
CBLN1	40	43.2	48.8	70.4	65.6	65.1	64.2	52.1	55.1	66.6	55.1	48.1	89.7	82.7	47.3
CORIN	0.7	26.7	22.5	45.9	50.7	17.7	27.9	11.8	3.7	38.3	42.9	40.8	43.4	87.2	95.9
FOXP1	77.7	61.2	56.6	54.6	51.6	52.5	68.2	70.3	51.8	59.2	58.4	52.8	60.1	80.3	69.2
GATA2	56.2	44.8	29.4	55.7	40.2	16.1	76.2	48.4	32.5	48.1	40.6	53.9	60.1	53.6	23.4
IGLON5	80	60.1	59.6	42.5	37.3	17.4	41.2	55.3	34.8	43.2	30.8	33.1	35.2	72.4	54.2
NPTX2	57.9	45.4	48.7	57.6	69.2	55.5	1.2	26.4	30.1	41.1	39.5	34.5	45.3	74.4	72.4
NTRK2	47.9	34	29.4	37.4	53.7	2.1	33.2	46.5	15.1	48.5	37.6	50.1	53.8	58.4	46.4
PRL	21.9	14.5	29.3	16.9	11.7	42.3	16.6	18.4	41.4	45.3	61.3	47.3	33.7	30.1	18.4
STEAP4	52.2	35.9	29	59.9	48.4	53.8	46.8	39.0	21.5	68.8	37.7	41.5	35.4	65.9	41.0

Continued

Gene name	mL_21-3	mL_22-1	mL_22-2	mL_22-3	mL_22-4	SNGII	MCF7	Endo.ca_1	Endo.ca_2	Cerv.ca_1	Cerv.ca_2	SiHa	HecI	LMS_1	LMS_2
ALX1	87.4	75.0	72.3	66.0	64.1	50.8	49.6	31.1	59.5	60.2	55.4	78.9	95.3	48.5	85
CBLN1	73.4	73.1	77.7	73.4	68.6	28.8	56.2	15.3	44.5	48.3	48.5	46.3	24	28	39.8
CORIN	56.1	39.0	61.3	47.9	60.8	3.6	90.4	0.2	3.4	0.1	0.1	20.5	97.1	55.6	53.5
FOXP1	77.2	70.4	71.3	65.8	70.6	59.4	44.1	2.9	4.2	0.2	0.6	98.3	63	39.5	78.7
GATA2	36.8	59.9	59.1	51.2	61.8	93.1	89.6	65.3	33.2	88	65.3	93.1	90.9	87.6	16
IGLON5	53.9	37.5	32.0	55.4	22.9	93.4	93.7	0.6	50	67.6	54.5	94.9	90.2	73.5	0.7
NPTX2	59.4	42.4	61.0	14.6	25.4	0.6	0.2	0	0	0	0	0	28.9	11.4	24.6
NTRK2	82.6	38.8	51.8	78.1	41.5	0.4	0.3	0	0	0	0	14.9	98.3	78.9	53.1
PRL	25.1	21.7	40.4	16.6	41.6	94.4	89.1	99.9	99.7	99.6	99.9	79.9	99.5	98.6	15.7
STEAP4	56.2	51.0	60.6	58.7	56.2	9.6	2.7	11.8	6.5	1.2	8.9	0.6	54.8	88.5	49.2

Continued

Gene name	LMS_3	LMS_4	LMS_5	LMS_6	LMS_7	LMS_8	LMS_9	LMS_10	LMS_11	LMS_12	SKN	RKN
ALX1	12	36.5	41.7	38.3	66.2	47.6	56.4	61	52.9	29.7	77.8	51.3
CBLN1	28.6	1.2	67.2	21.5	99.6	99.6	0.5	30	21.5	5	6.8	0.2
CORIN	0	20.7	0.4	28.4	0.3	26.6	0.2	26	28.9	2.9	47.2	90
FOXP1	34.3	0.1	44.9	0.6	66	0	7.4	0	0	0	15.5	11.6
GATA2	64.7	79	37.8	58.8	47.5	7.6	0.2	27.4	36.6	44.5	82	95
IGLON5	72.5	69.1	0.5	0.5	77.4	0.3	32.8	0	0	0	46.9	49
NPTX2	16.8	0.2	0.2	0	41.9	37.9	35.4	49.4	23	45.6	0	0
NTRK2	0.2	0.3	26.7	0.1	16	29.7	29	5.6	1.8	1.8	18.5	11.6
PRL	22.7	14.6	11.3	9.9	49.2	24	43.6	75.1	29.8	25.1	97.5	90.2
STEAP4	22.2	1	9.3	39.7	36.2	62.3	2.2	38.6	50.1	29.4	24	33.9

Continued

a

Genotype	Number of cases (case #)	Nucleotide change
Point mutation	1 case (#4)	Detail in Supplementary Figure S1 b
Normal	11 cases (#1-3, 5-12)	-

b

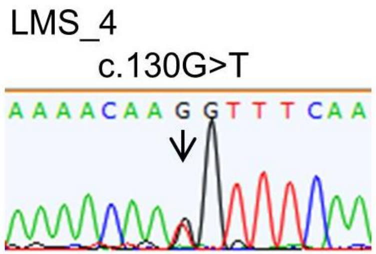

Supplementary Figure S1. Analysis of somatic mutations of *MED12* gene in the leiomyosarcoma specimens. (**a**) Summary of the *MED12* mutation analysis in 12 leiomyosarcoma specimens used in the study. (**b**) Sequencing chromatogram showing the point mutation in *MED12* in a leiomyosarcoma specimen. The point mutation is shown on the chromatogram. Mutated base is indicated by arrow.

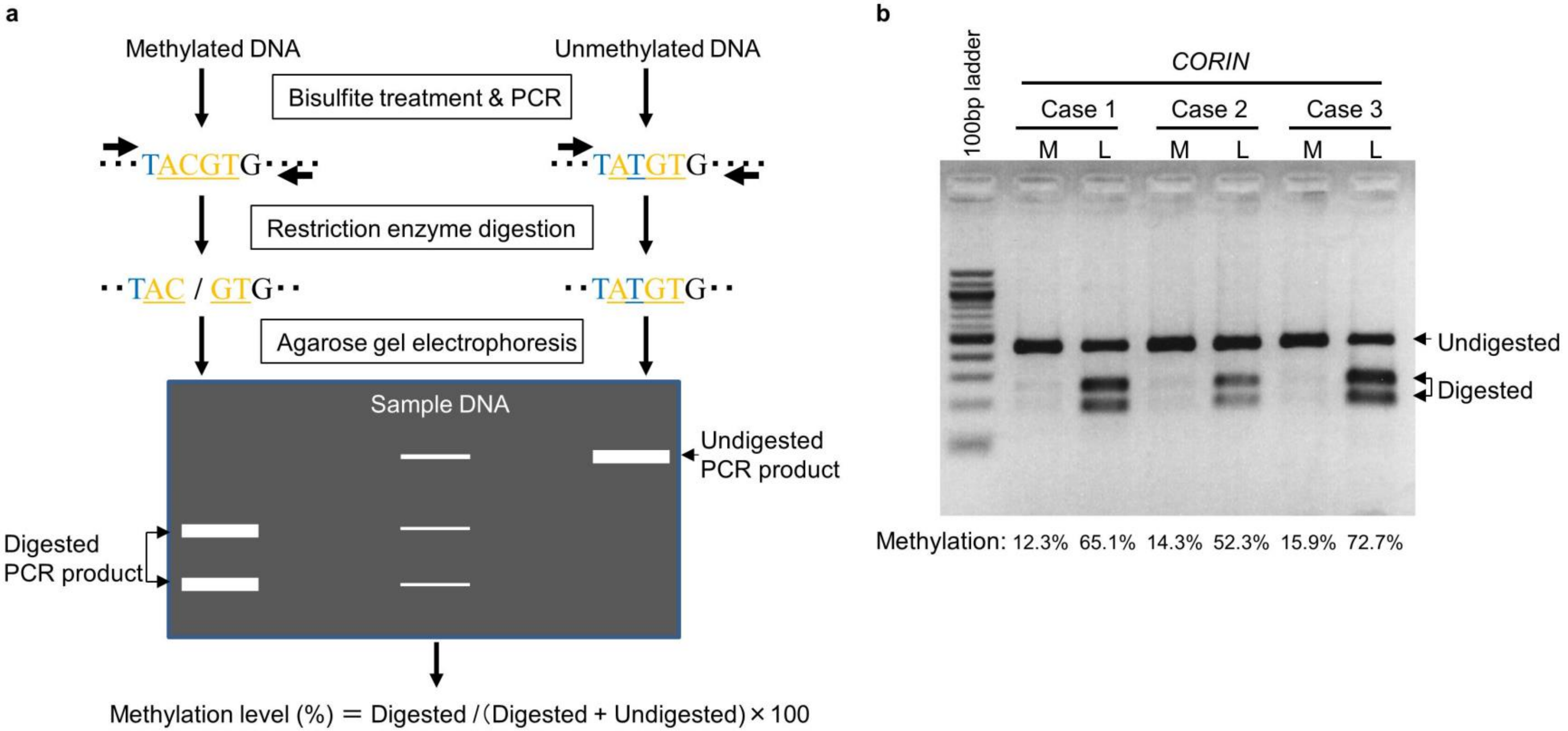

Supplementary Figure S2. COBRA method. (**a**) Outline of COBRA procedure. After sodium bisulfite treatment, PCR was performed. A part of the PCR products was treated with the restriction enzyme *Taq*I or *Hpy*CH4IV. The treated PCR products were electrophoresed by 3% agarose gel. PCR products from methylated DNA and unmethylated DNA are digested and undigested by the treatment with restriction enzymes, respectively. The intensity of the signals of the digested and undigested PCR products was measured by densitometry. Methylation levels (%) were calculated as the ratio of the digested PCR product in the total PCR product (digested + undigested). (**b**) Representative of agarose gel electrophoresis in COBRA method for *CORIN* gene and methylation levels (%) of three cases of uterine leiomyoma (L) and adjacent myometrium (M).
